# Complete Genome Sequence of *Lactiplantibacillus plantarum* VHProbi O10, Isolated from Kimchi

**DOI:** 10.1128/mra.00935-22

**Published:** 2022-10-26

**Authors:** Hongchang Cui, Jun Wang, Zhi Duan

**Affiliations:** a Qingdao Vland Biotech Group Co., Ltd., Qingdao, China; Queens College CUNY

## Abstract

Lactiplantibacillus plantarum strain VHProbi O10 is our proprietary probiotic that was isolated from kimchi soup. Here, we investigated the whole-genome sequence of this strain, which contains a chromosome and a plasmid.

## ANNOUNCEMENT

One milliliter of kimchi soup purchased from a supermarket was diluted in 99 mL of de Man-Rogosa-Sharpe (MRS; HopeBio catalog number HB0384-1) broth medium. Then, 1 mL of dilution was spread onto the MRS agar plate and incubated under aerobic conditions at 37°C to enrich bacteria. After 48 h, a white colony designated VHProbi O10 was picked up and identified as the species Lactiplantibacillus plantarum using 16S rRNA sequencing as described by Fu et al. (1). A combined sequencing strategy of Illumina HiSeq 2500 and the PacBio RS II single molecular real-time (SMRT) platform was used to sequence the genome of this strain. The strain was cultured in MRS broth medium under aerobic conditions at 37°C for 48 h for DNA extraction. The genomic DNA purification kit (Promega, USA) was used to extract DNA samples following the kit instructions. The Illumina library and the PacBio library used the same DNA samples. DNA was sheared into 400 bp for the Illumina library build using the TruSeq library preparation kit, and the prepared libraries were used for paired-end Illumina sequencing (2 × 150 bp). For the PacBio sequencing, DNA was sheared into 10-kb fragments and then purified, end repaired, and ligated with adapters using the SMRTbell Express Template Prep kit 2.0. The prepared libraries were sequenced on one SMRT cell following the PacBio recommendations. Finally, 7,623,164 raw reads and 7,501,080 clean reads were generated using the Illumina sequencing platform to reach a depth of 351.04-fold coverage, and 282,759 raw reads (*N_50_*, 231,611 bp) were generated using the PacBio sequencing platform. The Illumina raw reads were quality controlled using Sickle v1.33 (https://github.com/najoshi/sickle), and the clean reads were assembled into a scaffold using SOAPdenovo v2.04 (2). The PacBio raw reads were assembled into a scaffold using the hierarchical genome assembly process (HGAP) and canu v2.2 (3). Pilon v1.22 was used to check the PacBio assembly results using the Illumina reads (4). If there was an overlap at the two ends with 5,000 bp, one end of the overlap was cut off to circulate the sequences. The final assembly generated a seamless chromosome and a plasmid. Glimmer v3.02 (5), tRNA-scan-SE v2.0 (6), and barrnap v0.9 (https://github.com/tseemann/barrnap/) were used to predict the protein-coding genes, tRNA, and rRNA, respectively. CheckM v1.1.3 was used to check the quality of the assembly results with completeness of 99.07%, contamination of 2.78%, and heterogeneity of 0% (7). Genome annotations were conducted using the NCBI Prokaryotic Genome Annotation Pipeline (PGAP) v6.1 (8). Default parameters were used for all software unless otherwise specified.

The whole-genome sequence of *L. plantarum* VHProbi O10 contains a 3,247,797-bp circular chromosome and a 19,592-bp circular plasmid with GC contents of 44.49%. There were 2,947 protein-coding genes, 16 rRNA genes, 71 tRNA genes, 4 noncoding RNA (ncRNA) genes, and 40 pseudogenes in the genome.

### Data availability.

The whole-genome sequence of *L. plantarum* VHProbi O10 has been deposited in databases with GenBank accession numbers CP097163 and CP097164, SRA accession numbers SRR20760922 and SRR20760921, BioProject accession number PRJNA835513, and BioSample accession number SAMN28104611.
